# B Type and Complex A/B Type Epicatechin Trimers Isolated from *Litchi pericarp* Aqueous Extract Show High Antioxidant and Anticancer Activity

**DOI:** 10.3390/ijms19010301

**Published:** 2018-01-19

**Authors:** Yihui Gong, Fang Fang, Xin Zhang, Bin Liu, Honghui Luo, Zhen Li, Xuelian Zhang, Zhaoqi Zhang, Xuequn Pang

**Affiliations:** 1 State Key Laboratory for Conservation and Utilization of Subtropical Agro-bioresources/Guangdong Provincial Key Laboratory of Postharvest Science of Fruits and Vegetables/College of Horticulture, South China Agricultural University, Guangzhou 510642, China; gyhzgh@163.com (Y.G.); bin278083@foxmail.com (B.L.); hhluo@stu.scau.edu.cn (H.L.); xuelianzhang@scau.edu.cn (X.Z.); 2 College of Life Science, South China Agricultural University, Guangzhou 510642, China; fangfangdc@gmail.com (F.F.); zhang_ivy@163.com (X.Z.); 3 Research Institute of Food Science and Engineering Technology, Hezhou University Hezhou 542899, China; 4 Institute of Biomedicine, Guangdong Provincial Key Laboratory of Bioengineering Medicine, Biomedical Translational Research Institute, Jinan University, Guangzhou 510632, China; 810leezhen@sina.com

**Keywords:** Litchi fruit, aqueous pericarp extract, B-type epicatechin trimers, A/B-type epicatechin trimers, anticancer activity

## Abstract

Litchi (*Litchi chinensis* Sonn.) fruit is known for its rich source of phenolics. *Litchi pericarp* contains high levels of epicatechin that may form oligomers of various lengths. Except for several A or B type epicatechin dimers, other soluble oligomers have rarely been identified in the pericarp. Here, bioassay-guided column fractionation was applied to isolate bioactive phenolics from aqueous pericarp extract. A fraction (S3) was obtained by two rounds of Sephadex LH-20 column chromatography, and showed higher antioxidant activity and inhibition on the proliferation of human lung cancer cells (A549) than *Litchi anthocyanins*. S3 was further separated to isolate fractions P1–P4, which all showed higher antioxidant activity than vitamin C. P3 showed 32.9% inhibition on A549 cells at 30 μg/mL, higher than other fractions and *cis*-Dichlorodiamineplatinum (DDP, 0.5 μg/mL), but not as high as the combination of the four fractions. Using HPLC-Q-TOF-MS/MS, one B-type and complex A/B type epicatechin trimers were identified in P3; another B-type and two A/B-type trimers were identified in P4. P1 and P2, containing epicatechin and proanthocyanidin B2, respectively, showed no cell inhibition at 30 μg/mL. It is the first time that the two B type trimers of epicatechins (Litchitannin B1 and B2), have been found in Litchi species. The identified proanthocyanidins were detected in the pericarp of the young fruit, and the levels of the compounds decreased as the fruit developed, correlating to the decreasing patterns of the expression of *LcLAR* and *LcANR*, two key genes in the catechin biosynthesis pathway.

## 1. Introduction

Litchi (*Litchi chinensis* Sonn.) fruit has a bright red and attractive pericarp surrounding a white and translucent fleshy aril, having delicious and great nutritional value [1]. As recorded in the Compendium of Materia, an influential book in traditional Chinese medicine history, Litchi fruit has been used as an anodyne in hernia, ulcers, and for intestinal troubles for more than 1000 years. The fruit is rich in polyphenols; Brat et al. reported that its polyphenol content per edible part is second only to strawberries [2]. Recent medical reports have published that Litchi fruit possesses many bioactivities, such as hypoglycemic, anticancer, antibacterial, anti-hyperlipidemic, anti-platelet, and antiviral [3,4,5]. The Litchi industry in China is expanding and the yield per year reached 1.75 million tons in 2010 [6]. Some Litchi cultivars exhibit a high yield of fruits and are promising sources for bioactive compound extraction.

Litchi fruit contain different classes of natural products with varying structural patterns [3]. The major components of fresh *Litchi pericarp* extract were anthocyanins, proanthocyanidins, phenolic acids, and coumarins, etc., which are associated with high antioxidant activity and a lower incidence of cancers [7]. Anthocyanins from *Litchi pericarp* have been found to strongly inhibit linoleic acid oxidation and exhibit a dose-dependent free radical scavenging activity against DPPH radical [8]. Epicatechin, proanthocyanidin B2, proanthocyanidin B4, and the ethyl acetate fraction from *Litchi pericarp* were found to possess much higher stimulatory effects on splenocyte proliferation than that of rutin, while showed lower cytotoxicities to human breast cancer cell MCF-7 and human embryolic lung fibroblasts than paclitaxel [9]. Oligonol, produced by oligomerizing polyphenol polymers derived from dry lychee fruit and containing catechin-type monomers and smaller oligomers of proanthocyanidin, has been reported to exhibit beneficial bioactivities in many studies, and is expected to show favorable effects on various chronic diseases [10,11]. However, except for monomers or dimers of proanthocyanidin, longer water soluble oligomers have rarely been identified in *Litchi pericarp* extract, in contrast to the abundance of the monomers or dimers of proanthocyanidin in the pericarp [3]. The isolation of oligomers may be affected by the extraction strategy and the status of the pericarp tissue. Ethanol extraction of dry pericarp tissue has been applied for Litchi fruit polyphenol extraction [12], in which dry tissues usually contain highly polymerized proanthocyanidins that are practically insoluble in water. Other extraction/isolation strategies and investigations of the effect of tissue status/developmental stage may help to identify more bio-functional oligomers of proanthocyanidin in the *Litchi pericarp*.

Recently, combinatorial chemistry and bioassay-guided fractionation was suggested for the purification of pharmacologically active phytochemicals [13]. Based on this strategy, phenolic compounds were extracted and purified from freshly frozen *Litchi pericarp* by 0.1 M aqueous solution. A phenolic fraction obtained by Sephadex LH-20 chromatography was preliminarily confirmed to possess inhibition activity to human lung adenocarcinoma cells (A549) and high antioxidant activity. The fraction was further purified and a subsequent fraction, in which epicatechin (EC), and three dimers and six trimers of proanthocyanidins, were identified by LC-MS, possessed the anticancer activities. We then analyzed the accumulation profiles of four catechin-type compounds during fruit development and the expression patterns of relevant biosynthesis-related genes.

## 2. Results and Discussion

### 2.1. A Fraction from Litchi phenolic Extract Displayed Strong Inhibition on Cancer Cell Proliferation and Antioxidant Activity

**Figure 1:**
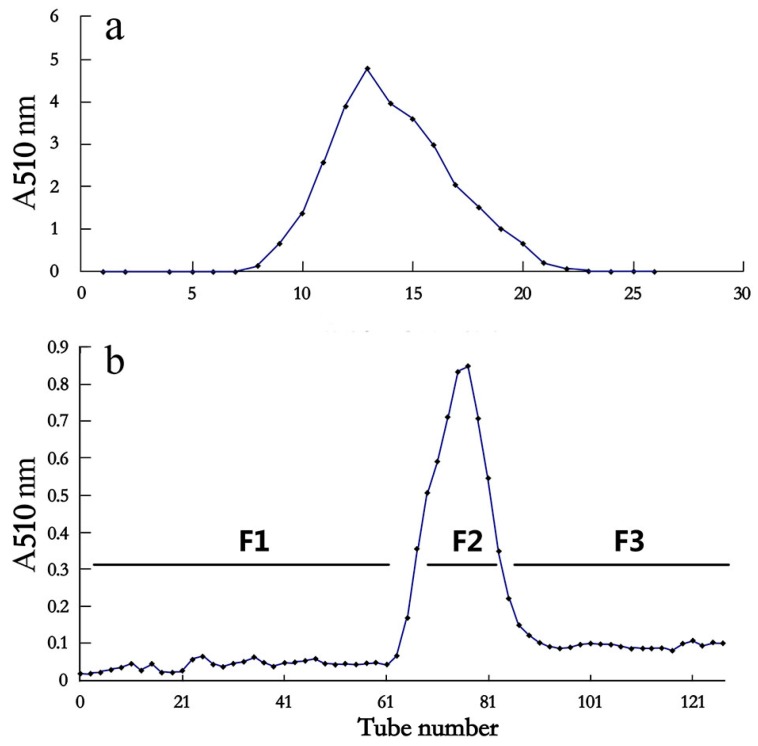
Separation of the aqueous extract of *Litchi pericarp* by column chromatography. (**a**) The crude aqueous extract was first loaded on the AmberliteXAD-7 resin column and eluted by methanol with 0.1% (*v*/*v*) HCl, and absorbance at 510 nm (A510) of the fractions was monitored; (**b**) The red fractions in (**a**), with high A510, were combined and separated by the Sephadex LH-20 column. F1 (tube number 1–60), F2 (61–91) and F3 (92–130) fractions were combined respectively according to their absorbance at 510 nm.

The phenolics in *Litchi pericarp* were extracted with 0.1 mol/L HCl aqueous solution. The extract was first purified by an Amberlite XAD-7 resin column, and the red fractions with absorbance at 510 nm (Figure 1a) were combined and concentrated for the next purification by the Sephadex LH-20 column. After separation by the Sephadex LH-20 column, the elution was divided into three fractions according to A510, namely F1, F2, and F3 (Figure 1b). F2, with high A510, contained a high amount of anthocyanins [14]. The fractions that were eluted before and after the anthocyanin fraction (F2) were combined, respectively, as F1 and F3. The three fractions were respectively freeze dried and aqueous relevant solutions were obtained.

**Figure 2:**
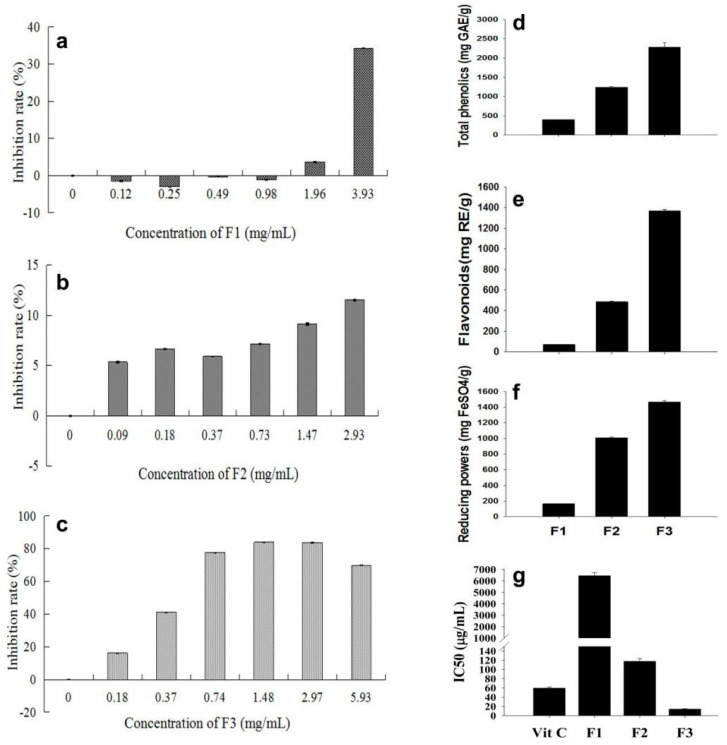
Anticancer and antioxidant activities of F1, F2, and F3 fractions. F1 (**a**), F2 (**b**), and F3 (**c**) fractions, as shown in Figure 1 b, were diluted in a series of concentrations, and their inhibition on the proliferation of human lung adenocarcinoma cells (A549) was determined; (**d**) Total phenolic contents expressed as gallic acid equivalents in the fractions; (**e**) Flavonoid contents expressed as rutin equivalents in the fraction; (**f**) Reducing powers expressed as FeSO_4_ equivalents of the fractions; (**g**) IC50 values of the fractions for DPPH radical scavenging activity with vitamin C (Vit C) as the reference. The values are the means of three measurements. Error bars indicate the SEM (Standard Error of Mean) of the values.

To measure the anticancer activity of the three fractions, the inhibition of a series of concentrations of the factions on the proliferation of human lung adenocarcinoma cells (A549) was measured. F3 showed around 80% inhibition of the cell proliferation at 0.74 mg/mL (Figure 2c). For F1, inhibition was not observed until the concentration was increased to 1.96 mg/mL, and 34% inhibition was detected for 3.93 mg/mL (Figure 2a). Around 12% inhibition was detected for F2 at 2.93 mg/mL (Figure 2b).

More than 1000 and 2000 mg gallic acid equivalence per gram (mg GAE/g) was detected for F2 and F3 factions (Figure 2d), respectively, indicating that the factions were mainly phenolic compounds. F2 and F3 were detected as 500 and 1400 mg rutin equivalence per gram (mg RE/g, Figure 2e), respectively, indicating that the compounds in F3 were mainly flavonoids. F2 and F3 showed a strong ferric reducing antioxidant power (FRAP), as indicated by 1000 and 1400 mg FeSO_4_ equivalence per gram, respectively (Figure 2f). F3 showed the strongest radical scavenging activity, as indicated by the IC50 values of 12.3 μg/mL to DPPH, lower than the value of vitamin C (59.45 μg/mL) (Figure 2g). Taken together, the F3 fraction showed a stronger reducing power and radical scavenging capacity than the other factions, including the F2 anthocyanin faction, and vitamin C.

Anthocyanin pigments exhibit a wide range of properties: antioxidant protection, and anti-inflammatory, antimicrobial, and anticarcinogenic activity, thus reducing the risk of cancer [15,16,17]. Faria et al. reported that some anthocyanin components in blueberry are effective in inhibiting cancer or induce mechanisms that may kill cancer cells and inhibit tumor invasion [18]. Cyanindin-3-*O*-rutiside was the major anthocyanin identified in *Litchi pericarp* [14,19]. The present study indicated that F3 possesses even much higher anticancer and antioxidant activities than the F2 anthocyanin fraction (Figure 2).

### 2.2. The Fraction with High Anti-Cancer Activity was Further Separated, and a Sub-Fraction S3 Was Isolated with High Activity

**Figure 3:**
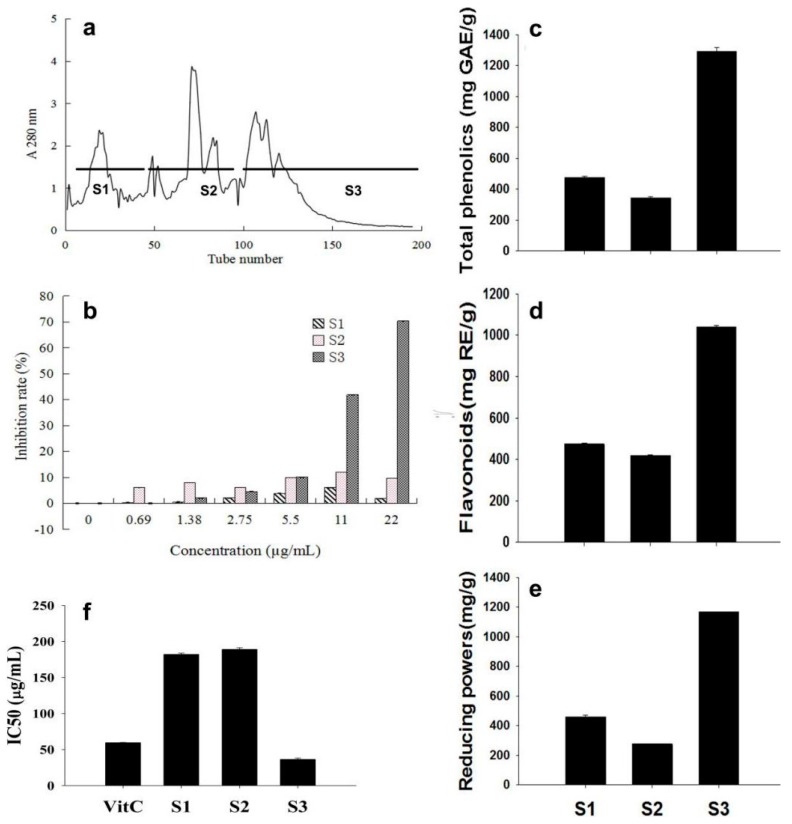
Separation of F3 fraction and the anticancer and antioxidant activities of the sub-fractions, S1, S2, and S3. (**a**) F3 fraction (Figure 1b and Figure 2), which was found exhibit high anticancer activity, was further separated by the LH-20 column, and three sub-fractions, S1 (1–39), S2 (40–96), and S3 (97–195), were obtained. (**b**) Anticancer activities of S1, S2, and S3 were determined as described in Figure 2a–c. Contents of total phenolics (**c**), flavonoids (**d**), and the reducing powers (**e**) and IC50 values for DPPH radical-scavenging activities (**f**) were detected and expressed as described in Figure 2. The statistic details of the data are as described in Figure 2.

F3 was further separated by the LH-20 column and three sub-fractions were obtained: S1, S2, and S3 (Figure 3a). Similar to the previous separation, the three sub-fractions were respectively freeze dried and aqueous solutions were obtained.

The inhibition of the three sub-fractions on the proliferation of A549 cells was analyzed. S3 showed clear dose dependent inhibition on the cell proliferation; 41.6% and 70.35% inhibition were detected at 11 and 22 μg/mL, respectively. Compared to S3, significantly weaker inhibition was found for S1 and S2, with the highest inhibition of 6.02% and 12.14%, respectively, at 11 μg/mL (Figure 3b). S1, S2, and S3 were determined as 474, 342, and 1292 mg GAE/g, respectively (Figure 3c); 472, 418, and 1041 mg RE/g of flavonoids (Figure 3d). S3 exhibited higher FRAP than S1 and S2, as indicated by 460, 276, and 1169 mg FeSO_4_/g for S1, S2, and S3, respectively (Figure 3e). IC50 values of S1, S2, and S3 were determined as 182, 189, and 36 μg/mL, respectively (Figure 3f), for which only S3 showed a lower IC50 value than vitamin C (59.45 μg/mL).

### 2.3. The Sub-Fraction with High Anti-Cancer Activity Was Further Separated and a Fraction Contained a B-Type Trimer Proanthocyanidin Showed High Antioxidant and Anticancer Activities

**Figure 4:**
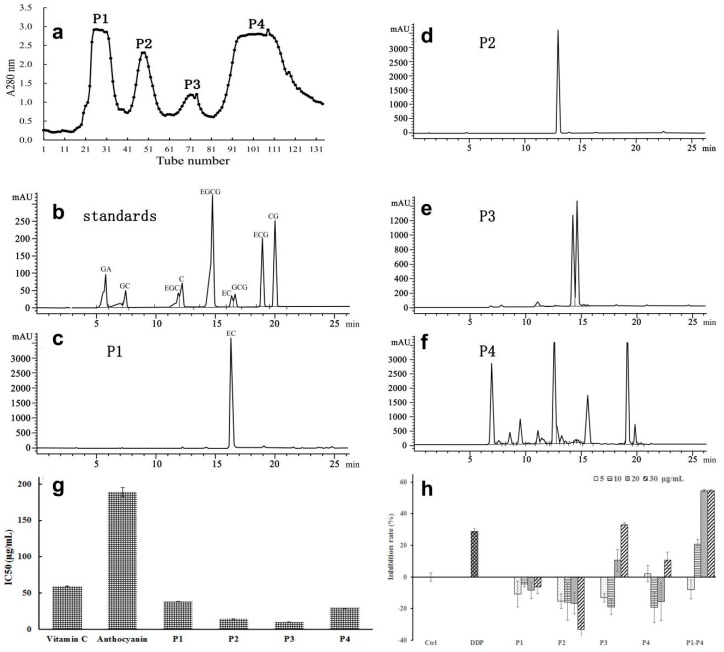
Separation and identification of the compounds in S3 fraction. S3 fraction (Figure 3) was further separated by Sephadex LH-20 chromatography and A280 peaks were monitored and combined as P1, P2, P3, and P4 (**a**). (**b**–**f**) Identification of the compounds in P1–P4 by HPLC analysis. (**b**) HPLC separation of the standards of catechin-type monomeric compounds. HPLC analysis of P1, 2, 3, and 4 (described in (**a**)) is shown in (**c**–**f**), respectively. Only EC (Epicatechin) was identified in P1 based on the comparison with the standards (**b**). Unknown compounds were also detected in P2 (**d**), P3 (**e**), and P4 (**f**). (**g**) Comparison of IC50 values for DPPH radical-scavenging activities of the reference Vit C, *Litchi anthocyanin* (cyanidin-3-rutinside) that was purified by LH-20 column, and the fractions P1–P4, expressed as micrograms gallic acid equivalents per milliliter (μg/mL) of the fractions. (**h**) Anticancer activities of the fractions P1–P4 at 5–30 μg/mL were determined as described in Figure 2, with 0.5 μg/mL cis-Dichlorodiamineplatinum (DDP) as the reference. The activity of the combination of an equal amount of P1–P4 was also detected. The statistic details of the data are as described in Figure 2.

To further identify the compounds in S3, the faction was further separated by the LH-20 column. Four major elution peaks were obtained and combined respectively as P1, 2, 3, and 4 for HPLC analysis (Figure 4a). One major compound was detected in P1 and P2 fractions respectively, while two and six major compounds were detected in P3 and P4 fractions respectively (Figure 4b–d). Compared to retention times of the standards, only the compound in P1 was identified as (−)-epicatechin (EC) (Figure 4c). P1–P4 fractions exhibited higher radical scavenging activities than Vit C and *Litchi anthocyanin* (cyanidin-3-rutiside), as indicated by the IC50 values of 18.68, 14.48, 10.14, and 29.41 μg/mL for P1–P4, respectively (Figure 4g), compared to values of 59.45 and 189.35 μg/mL for vitamin C and cyanidin-3-rutiside, respectively. Concentrations of 5–30 μg/mL for the P1-4 fractions were tested for the inhibition of the proliferation of A549 cells with the reference of cis-Dichlorodiamineplatinum (DDP). No inhibition activity was found for P1 or P2 at 30 μg/mL. P3 displayed a dose dependent inhibition on the cell proliferation, with 32.9% inhibition at 30 μg/mL, which is higher than the 28.9% inhibition seen for DDP (0.5 μg/mL). Inhibition of 10.5% was detected for P4 at 30 μg/mL, which was lower than both P3 and DDP (Figure 4h). Interestingly, the combination of P1–P4 increased the inhibition effect, with 54.4% inhibition at 20 μg/mL, which is significantly higher than that of DDP.

**Figure 5:**
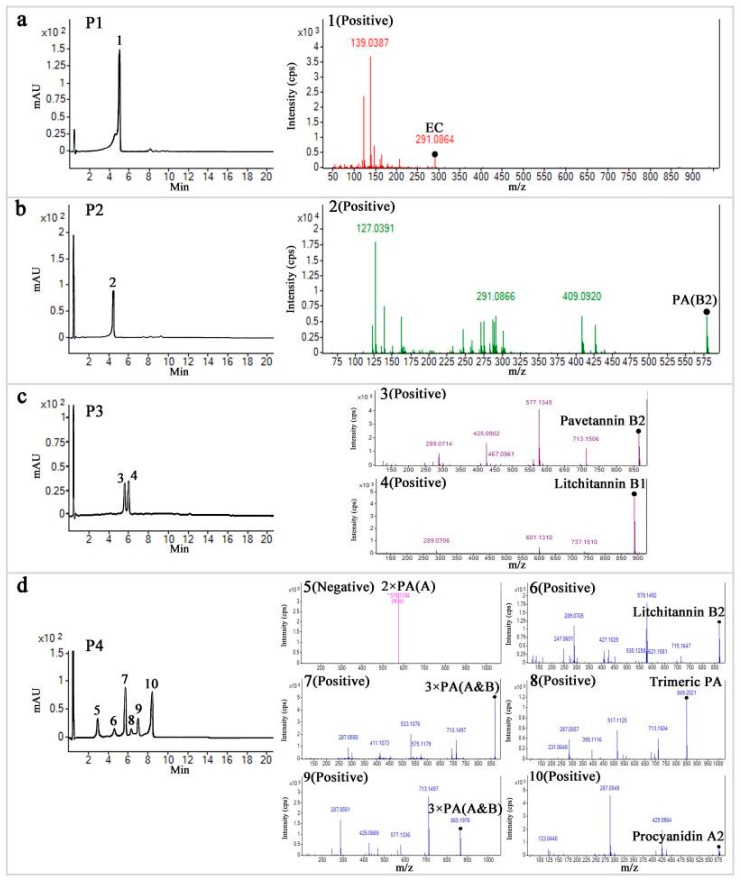
LC-MS separation and MS/MS spectra of the compounds in fractions P1–P4. (**a**–**d**) LC-MS/MS profiles of the compounds in P1–P4, as shown by the absorbance at 280.40 nm and relevant MS/MS spectra for the peaks identified by DAD. The compound peaks in the chromatography profiles were numbered from 1 to 10 for the fractions P1 to P4. High resolution MS/MS spectra (Q-TOF) in positive mode for the compounds are shown next to the chromatography profiles. No positive mode was achieved for compound **5**, and the MS spectrum in negative mode is shown. Brief names of the compounds, tentatively identified in the metlin database (https://metlin.scripps.edu), were shown with the spectra of the compounds (full names are shown in Table 1).

P1–P4 fractions were also analyzed by LC-MS to identify compounds in the fractions (Figure 5 and Table 1). The negative and positive *m/z* for the compound in P1 (compound **1**) was well correlated to the mass of epicatechin, which was also confirmed by HPLC (Figure 4c), and the major phenolics identified in *Litchi pericarp* [20,21]. The negative and positive *m/z* of the compound in P2 (Compound **2**) was 579 and 577, together with the fragment ions in MS/MS, and the similar HPLC profile detected for an ethyl acetate fraction of *Litchi pericarp* containing proanthocyanidin B2 and epicatechin [22] indicates that the compound is a B-type procyanidin, a dimer of proanthocyanidin B2.

The two compounds (Compound **3** and **4**) in P3 had molecular weights consistent with molecular weights of procyanidin trimers. The fragment ions in MS/MS and the deduced molecular formula indicate that compound **3** is a trimer of proanthocyanidin B2A, and **4** is a trimer of proanthocyanidin B. The mass spectra detected in P4 are consistent with the molecular weight of dimers and trimers of procyanidins, with two A-type dimers of proanthocyanindin (**5** and **10**), two trimers of complex proanthocyanidin AB (**7** and **9**), a trimer of proanthocyanidin B (**6**), and a trimeric proanthocyanidin with an afzelechin or epiafzelechin unit (**8**). Based on NMR and LC-MS analysis, proanthocyanidin A1, A6, and A2 (C_30_H_24_O_12_, mass: 576.1) were identified in the seeds, leaves, and pericarp of Litchi [23,24]; proanthocyanidin B2 and B4 were found in the ethyl acetate fraction of ethanol *Litchi percarp* extract (C_30_H_26_O_12_, mass: 578.1) [22]; and two A-type trimeric proanthocyanidins (Litchitannin A1 and A2 molecular formula C_45_H_34_O_18_, mass: 862.1) and a trimer of complex proanthocyanidin AB (aesculitannin A, molecular formula C_45_H_36_O_18_, mass: 864) were identified in the ethanol extract of Litchi seeds [23]. A-type dimers and trimers of proanthocyanidin possess one and two doubly bonded interflavanoid linkages, respectively, which are distinguished from B-type dimers and trimers by their molecular formula and masses. This can be applied for the judgment of the polymerization types of the oligomers of proanthocyanidin [23]. Accordingly, based on the *m/z* and *ms/ms* of the ions and fragment ions, and the information on the proanthocyanidin dimers and trimers published in Litchi in previous studies [3], three dimers and six trimers of proanthocyanidin were identified in the fractions from aqueous Litchi pericarp extract, by using column chromatography combined with a bio-assay strategy. It is the first time that two trimers of proanthocyanidin B (C_45_H_38_O_18_, mass: 866.2) compounds **4** and **6** have been reported in Litchi [3], which were accordingly designated as Litchitannin B1 and B2. Interestingly, most of the trimeric proanthocyanidins identified in Litchi seed ethanol extract were A-type [23], while in the present study, no A-type trimers were found; in contrast, two B-type and three complex A/B type trimers were identified in the aqueous extract of pericarp.

It was suggested that the inhibition activities against a human prostate cancer cell line (PC-3) were associated with the degree of oligomerization of epicatechin, and that the antioxidant activities are dependent on the number of hydroxyl groups in their molecular structures [25,26]. P3 contained compounds **3** and **4**, trimers of proanthocyanidin B2A and B (Litchitannin B1), showing the highest antioxidant and cytotoxitic activities among the four fractions. These results could be due to these compounds being the longest oligomers in the fractions, and the fact that Litchitannin B was a B-type trimer proanthocyanidin with more hydroxyl groups than the A-type counterparts. The fraction showed around 40% inhibition on A495 cells at 30 μg/mL, and the cytotoxicity may be higher than *Litchi proanthocyanidin* B2, whose IC50 to MCF-7 cells was 99 μg/mL (versus the reference paclitaxel of IC50 value less than 25 μg/mL) [9]. In the present study, the combination of P1–P4 significantly increased cytotoxicities to the cancer cells, in agreement with the suggestion by other studies that additive or synergistic effects of bioactive phyto-constituents might be responsible for the concerned pharmacological function rather than purified ones [13]. Litchitannin B1 and B2, while of the same molecular weight, were separated by HPLC, indicating that they are of different molecular structures. The molecular structures of the compounds are required to be further characterized.

### 2.4. Contents of Catechins and Proanthocyanidins in Litchi pericarp during Fruit Development

**Figure 6:**
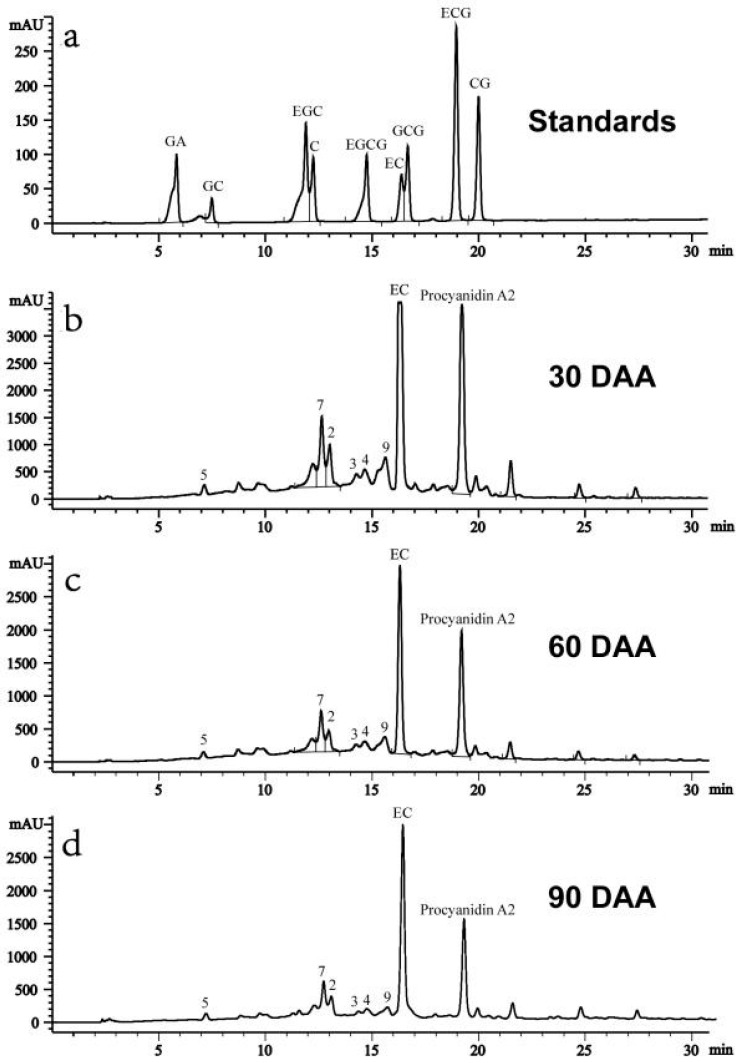
**Figure** **6.** HPLC profiles of and proanthocyanidin compounds of the *Litchi pericarp* extract from the fruit at different developmental stages. (**a**). HPLC separation of the standards of catechin-type monomers. (**b**–**d**). HPLC analysis of the proanthocyanidin compounds in the *Litchi pericarp* of the fruit harvested at 30 days after anthesis (DAA) (**b**), at 60 DAA (**c**), and 90 DAA (**d**). The peaks were identified in the samples as described in Figure 4 and 5. The statistic details of the data are as described in Figure 2.

Two catechin-type monomers and eight proanthocyanidins identified in P1–P4 fractions were assigned to the peaks of HPLC profiles of *Litchi Pericarp* phenolic extract, with nine catechin-type monomers and compounds **1**–**10** as references (Figure 6), including catechin, epicatechin (**1**), Proanthocyanidin B2 (**2**), Pavetannin (**3**), Litchitannin B1 (**4**), Proanthocyanidin A (**5**), trimers of complex proanthocyanin A and B (**7** and **9**), and procyanidin A2 (**10**). The contents of compounds **6** and 8 in the total phenolic extract might be too low to be identified. The retention time of procyanidin A2 (10) was very close to that of epicatechin-3-gallate (ECG), and was considered as ECG by Zhang et al. [20]. The peak in the HPLC profile was confirmed not to be ECG, based on no peak enhancement after the addition of ECG standard to the samples and on [M − H]^−^ at *m/z* 575 as detected by LC-MS analysis of the HPLC purified compound (data not shown).

**Figure 7:**
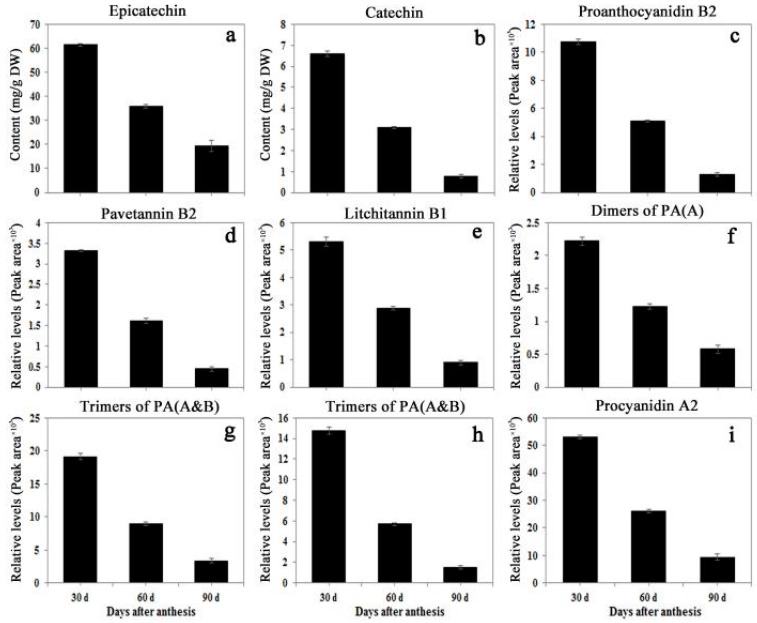
Contents of catechins and proanthocyanidins in *Litchi pericarp* during fruit development. Contents of epicatechin (**a**), catechin (**b**), proanthocyanidin B2 (**c**), pavetannin B2 (**d**), Litchitannin B1 (**e**), dimers of proanthocyanidin A (**f**), trimers of complex proanthocyanidin A and B (**g**), trimers of complex proanthocyanidin A and B (**h**), and procyanidin A2 (**i**) in the samples as described in Figure 6 and Table 1 were detected by HPLC and LC-MS/MS. The contents of EC and C during fruit development were calculated according to standard curves, while the relative levels of the six proanthocyanidins were expressed as the peak area. The statistical details of the data are as described in Figure 2.

The contents of EC and C during fruit development and storage at 20 °C after harvest were calculated according to the standard curves C, while the relative levels of the eight proanthocyanidins were expressed as the peak area (Figure 7). In agreement with the data by Zhang et al. [20], *Litchi pericarp* contained high levels of epicatechin compared to other compounds, with more than 60 mg/g DW in the pericarp of young fruit at 30 days after anthesis (DAA), while catechin content was around 1/10 of the epicatechin content. The content of all the identified compounds decreased as the fruit developed from 30 to 90 DAA, and the contents continuously decreased after harvest. On day 4 after harvest, the fruit turned brown and only the compounds of a higher concentration, such as EC (**1**), procyanidin A2 (**10**), and trimer of complex proanthocyanidin A/B (**7**), could be detected (Figure S1). The result was consistent with the profiles found in strawberry [27,28] and grape [29] specimens during the development stages. The decreasing contents of compounds during the later developmental stages might be due to degradation or transformation into other compounds.

### 2.5. Expression of Catechin and Anthocyanin Biosynthesis Related Genes in Litchi pericarp during Fruit Development

**Figure 8:**
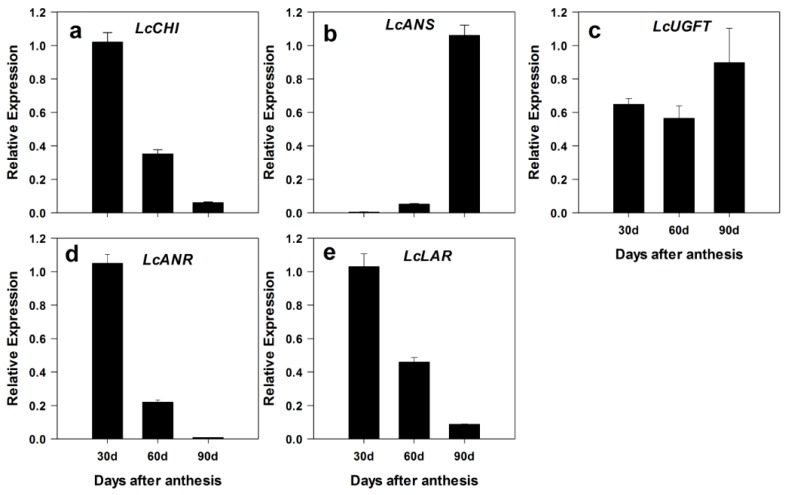
Relative expression levels of anthocyanin and catechin biosynthesis pathway genes. The gene expression levels in the pericarp samples collected during fruit development were analyzed by quantitative real-time PCR. *LcActin* (HQ615689) gene was used to normalize the expression levels of the genes under identical conditions. The relative expression levels of *LcCHI*, *LcANS*, *LcUGFT*, *LcANR*, and *LcLAR* were shown in (**a**–**e**). The statistical details of the data are as described in Figure 2.

The transcription levels of the genes that encode the key enzymes functioning in anthocyanin and catechin biosynthesis pathways were detected during fruit development by using real time qRT-PCR (Figure 8). Chalcone isomerase (CHI) is one of the key enzymes involved in flavonoid biosynthesis, contributing to the accumulation of flavonoid components during fruit maturation [30,31]. Anthocyanidin synthase (ANS) is one of the key enzymes in the biosynthesis of both anthocyanins and proanthocyanidins [32,33,34]. UDP-glucose: anthocyanidin 3-*O*-glucosyltransferase (UFGT) catalyzes the transfer of the glucosyl moiety to the 3-hydroxyl group of anthocyanidins [35]. The UGFT was the final enzyme in the anthocyanin pathway and was found to play an important role in the anthocyanin accumulation in Litchi [19,36]. Leucoanthocyanidin reductase (LAR) and anthocyanidin reductase (ANR) are both key enzymes of the branch of the catechin biosynthesis pathway [37,38]. They are responsible for the production of (+)-catechin and (−)-epicatechin, respectively [38,39].

High levels of *LcLAR*, *LcANR*, and *LcCHI* were detected at 30 DAA in the pericarp. At 90 DAA, relatively high transcription levels of *LcANS* and *LcUFGT* were recorded, while the expression levels of *LcLAR*, *LcANR*, and *LcCHI* declined to low levels. These results indicated that the decreasing expression levels of *LcLAR* and *LcANR* in the pericarp were consistent with the changes of catechin contents. Correlating to *LcLAR* and *LcANR* expression patterns, high contents of catechins were recorded in young Litchi fruit. The result was consistent with the data reported in strawberry [40] and grape [29] specimens. Gagné et al. also found that the LAR and ANR activity gradually decreased during development [41]. The expression levels of *LcANS* and *LcUFGT* were coordinated with rapid anthocyanin accumulation during the fruit maturation stage.

In conclusion, two B-type and three complex A/B type epicatechin trimers were isolated from the aqueous Litchi pericarp extract and showed a high cytotoxicity to A495 cells and antioxidant activities. The compounds highly accumulated in the young fruit and degraded with fruit development.

## 3. Materials and Methods

### 3.1. Plant Materials

Litchi (*Litchi chinensis* Sonn. Cv. Huaizhi) fruit were harvested from the Institute of Fruit Tree Research, Guangdong Academy of Agricultural Sciences in 2013–2014 during three developmental stages. The Litchi fruit of a uniform size and color were collected at 30, 60, and 90 days after anthesis (DAA) and the pericarp of the fruit was sampled. For the analysis of change in the postharvest fruit and phenolic extraction, mature fruit at 90 DAA was harvested and divided into two groups. The pericarp of the first group (around 100 kg) was peeled for phenolic extraction and purification. The fruit of the other group was stored in a controlled environment room at 20 ± 1 °C and about 70% relative humidity for six days, and the pericarp was sampled on day 0, 2, 4, and 6. All the sampled pericarp was quickly frozen in liquid nitrogen and stored at −80 °C for further analysis.

### 3.2. Extraction and Purification of Litchi pericarp Phenolics

Frozen *Litchi pericarp* (100 g) was blanched with 1 L of 0.1 mol/L HCl aqueous solution overnight. All extracts were combined and filtered through two layers of microcloth. The filtrate was then subjected to Amberlite XAD-7 resin (Sigma-Aldrich, Saint Louis, MO, USA) column chromatography according to Zhang et al. [14]. Red fractions with high absorbance at 510 nm (A510) were pooled and concentrated by removing the methanol at 40 °C in a rotary evaporator (LABOROTA 4002 Heidolph, Schwabach, Germany).

The concentrated solution was loaded onto a Sephadex LH-20 column (1 × 60 cm; Sigma-Aldrich, Saint Louis, MO, USA), eluted with aqueous 1% (*v*/*v*) formic acid with a methanol linear gradient (0–100% [*v*/*v*]) at a flow rate of 24 mL/h, and the elutes were collected using a fraction collector (3 mL per tube). Based on absorbance values at 510 nm (A510), three fractions, F1, F2, and F3, were pooled and concentrated by a rotary evaporator (LABOROTA 4002 Heidolph, Schwabach, Germany) at 40 °C. F2, with high A510, contained a high amount of anthocyanins [14]. The fractions eluted before and after the anthocyanin fraction (F2) were combined, respectively, as F1 and F3.

The F3 fraction was further separated by the LH-20 column using the method described above, and three sub-fractions were pooled based on A280 peaks, S1 (1–39), S2 (40–96), and S3 (97–195). The fractions were concentrated by a rotary evaporator at 40 °C and then dissolved in water. The solutions were used for the analysis of antioxidant and anticancer activity. S3 was further separated by using the LH-20 column, with a methanol linear gradient (90–100% [*v*/*v*]) at the same flow rate and collection as described above for 36 h. Four fractions (P1–P4) were pooled based on A280 peaks and concentrated for HPLC analysis.

### 3.3. Analysis for Total Phenolic Compounds

The total phenolic contents in respective fractions were estimated using a modified colorimetric Folin-Ciocalteu method [42]. Briefly, diluted fraction (0.25 mL) was added to deionized water (1.0 mL) and Folin-Ciocalteu reagent (0.25 mL). After 5 min, 7% (*v*/*w*) sodium carbonate solution (2.5 mL) was added, and the mixture was kept for 90 min at room temperature before measurement of the absorbance at 760 nm. The measurement was compared to a standard curve of gallic acid solutions and expressed as milligrams of gallic acid equivalents (GAE) per gram, mg GAE/g.

### 3.4. Determination of Total Flavonoid Content

The flavonoid contents of the fractions were measured using a modified colorimetric method [43]. Briefly, the fraction (0.25 mL) was added to distilled water (1.25 mL) and 5% (*v*/*w*) sodium nitrite solution (75 μL). After standing for 5 min, 10% (*v*/*w*) aluminum chloride (0.15 mL) was added to the solution and allowed to react for 6 min. Then, 1.0 mol/L sodium hydroxide (0.50 mL) was added, and the mixture was diluted with an equal amount of distilled water. The absorbance of the mixture at 510 nm was measured and compared to a standard curve of rutin. The flavonoid content was expressed as milligrams of rutin equivalents (RE) per gram, mg RE/g.

### 3.5. Ferric Reducing Antioxidant Power (FRAP) Assays

The FRAP assay measures the reducing potential of an antioxidant reacting with a ferric tripyridyltriazine (Fe^3+^-TPTZ) complex and producing a colored ferrous tripyridyltriazine (Fe^2+^-TPTZ). Total antioxidant power was determined using the FRAP assay [44], with slight modifications. The FRAP reagent was prepared by mixing 300 mmol/L acetate buffer (pH 3.6), 10 mmol/L TPTZ solution in 40 mmol/L HCl, and 20 mmol/L FeCl_3_ solution at a volume ratio of 10:1:1, and preheated to 37 °C before use. Sample solution (1 mL) with an appropriate phenolic concentration adjusted by methanol was mixed with 1.8 mL FRAP reagent. After incubation under darkness at room temperature for 10 min, the absorbance of the mixture was measured at 593 nm and compared to a standard curve of FeSO_4_. The FRAP antioxidant activity was expressed as mg FeSO_4_/g.

### 3.6. DPPH Radical Scavenging Activity

The DPPH radical-scavenging activities of the fractions were evaluated by modification of method [45]. Briefly, 20 μL of a series of the diluted fractions was mixed separately with 90 nmol/L methanolic solution of DPPH radical to a final volume of 1 mL. The disappearance of the DPPH radical was monitored by the decrease in absorbance at 515 nm, which was recorded after 0, 1, 2, 3, 4, and 5 min, and subsequently every 5 min up to 120 min, during which time the radical was stable. The scavenging activities were expressed as μg of the fractions required to decrease the initial concentration of DPPH radical by 50% (IC50, μg/mL).

### 3.7. Inhibition Activity Assay on Cancer Cells

The effect of the *Litchi pericarp* fraction on the proliferation of human lung adenocarcinoma cells (A549) was measured using the Cell Counting Kit-8 (CCK-8) (Sigma-Aldrich, Saint Louis, MO, USA) according to the manufacturer’s instructions. A549 cell line was kindly presented by Prof. Changliang Shan, Biomedical Traslational Research Institute, Jinan University, Guangzhou, China [46]. Briefly, the cells were seeded in flat bottomed plates (Greiner Bio-One, Kremsmuenster, Austria) with 5000 cells per well. Cells were incubated at 37 °C for 24 h in an environment with 5% CO_2_. Then, the fractions at a series of diluted concentrations were added for 48 h, followed by replacing old medium with 100 μL fresh medium containing 10 μL of CCK-8 solution. The plate was read at 450 nm and 630 nm using a spectrophotometric plate reader (CLARIOstar, BMGLabtech, Ortenau, Germany).

The cell inhibition ability was determined relative to the control using the following equation:

Cell inhibition ability (%) = [absorbance of control (450/630 nm) − absorbance of sample (450/630 nm)]/absorbance of control (450/630 nm) × 100%

### 3.8. HPLC-ESI-MS/MS Analysis of Fractions P1–P4

Separation of the compounds in the fractions of P1–P4 was performed in an Agilent 1200 Series HPLC system (Agilent Tech., Santa Clara, CA, USA). The fractions were injected into ZORBAX Eclipse XDB C18 (5 μm, 150 × 4.6 mm, Agilent Tech., Wilmington, DE, USA), and were separated with a linear gradient of 0.2% (*v*/*v*) formic acid and methanol over 45 min at a flow rate of 1 mL/min. The injection volume was 15 μL. The temperature of the column was 25 °C and the monitoring wavelength was recorded at 280 nm. The peaks of catechin-type compounds were identified by comparison with the retention time of the standards including (−)-epicatechin (EC), (−)-epicatechin-3-gallate (ECG), (−)-epigallocatechin gallate (EGCG), (+)-catechin (C), and (−)-catechin gallate (CG) (all from Sigma).

HPLC-DAD-ESI-MS/MS was performed in a UPLC1290-6540B Q-TOF (Agilent Tech., Singapore). coupled with a 6540UHD Q-TOF ESI Mass spectrometer (Agilent Tech., Singapore). The chromatographic separation was achieved on an Agilent eclipse plus 50 × 2.1 mm, 1.8 μm column. Mobile phases were acetonitrile (A) and formic acid 0.2% in water (B), at a flow rate of 0.3 mL/min. The mobile phase A gradient ranged from 5% to 60% in 35 min. The MS/MS detection of proanthocyanidins was carried out under the following optimized conditions: electrospray positive ionization mode, 10–30 V of collision energy, 4000 V of ionization voltage, and drying gas temperature at 300 °C. Nitrogen was used as the nebulizing gas (60 psi) and the fragmentor voltage was 130 V. Negative mode was also used in order to confirm the molecular weight and MS/MS profiles. The identification of the compounds was based on the searching of *m*/*z* values of the compounds in Metlin databases (https://metlin.scripps.edu/) and the fragment ions of MS/MS in the databases of Chemspider (http://www.chemspider.com/) and Pubchem (https://pubchem.ncbi.nlm.nih.gov/). The published Litchi metabolites also served as a reference [3].

### 3.9. Determination of the Contents of Catechin-Type Compounds and Proanthocyanidins by HPLC

A total of 0.1 g of freeze dried pericarp was ground into powder and extracted with 1.5 mL of *n*-hexane (with 3% [*v*/*v*] formic acid and 1% [*w*/*v*] butylated hydroxytoluene [BHT]) to remove fat and chlorophyll by shaking for 10 min. After centrifugation of 10,000 *g* for 10 min at 4 °C, the *n*-hexane was decanted and removed completely under reduced pressure. The phenolics were extracted with 1.5 mL methanol with 3% (*v*/*v*) formic acid and 1% (*w*/*v*) BHT. After vortexing (2 min) and sonication at 0 °C for 30 min, the samples were centrifuged 10,000 *g* for 10 min at 4 °C and the supernatant was then filtered by using a 22 μm polyvinylidenedifluoride membrane (ANPEL Scientific Instruments, Shanghai, China). The residue was re-extracted using the above-mentioned procedure, and the supernatant was combined with the first one [47]. Separation of catechin-type compounds and proanthocyanidins by HPLC was as described above. The peaks of the proanthocyanidins were identified by comparison with the retention times of the proanthocyanidins identified by LC-MS as described above. The contents of individual catechin-type compounds in the extraction were calculated according to the standard curves and the relative levels of the proanthocyanidins were expressed as the peak area.

### 3.10. Gene Expression Analysis

The total RNA was extracted from *Litchi pericarp* by using the RNAOUT kit (Huayueyang, Beijing, China) and first strand cDNA was synthesized with PrimeScript reverse transcriptase (TakaRa, Otsu, Japan). Specific primers for the catechin and anthocyanin biosynthesis genes were designed by primer 5 and are shown in Table 2. The specificity of primers was detected by the melting curve, analyzing the correct genes sequencing, and exhibited a PCR efficiency of about 90%. The transcription levels of the genes were analyzed using quantitative real-time PCR (qRT-PCR), with the SYBR Green PCR Supermix (TOYOBO, Osaka, Japan) and Bio-Rad CFX96 Real-Time PCR system according to the manufacturers’ instructions. PCRs contained 10 μL of SYBR Green PCR Supermix, 0.25 μL of each forward and reverse primer (100 nmol/L), 2.0 μL of cDNA template, and 7.5 μL of RNase-free water. *LcActin* (HQ615689) was used as a reference gene. Data analysis was calculated by the 2^−ΔΔ*C*t^ method [48]. All experiments measured three biological replicates.

### 3.11. Statistics

Data were collected from three independent extractions or measurements and values were described as means ± standard error of mean (SEM). All statistical analysis was performed by using SPSS17.0 software (SPSS Inc., Chicago, IL, USA).

## Figures and Tables

**Table 1:** The compounds identified in P1–P4 fractions by LC-MS/MS.

Peak	Compound Number	Mode	Mass	*m/z*	* Molecular Formula	Auto MS-MS Fragment Ions	* Tentative Identification
P1	1	pos	290.0792	291.0869	C_15_H_14_O_6_	139.0388; 123.044	EC (Epicatechin) [20,21]
		neg	290.1537	289.1464		245.0816; 125.0243	
P2	2	pos	578.1425	579.1498	C_30_H_26_O_12_	127.0391; 291.0866; 409.0920	PA (Proanthocyanidin) B2 [22]
		neg	578.1418	577.1345		125.0244; 289.0713; 407.0778	
P3	3	pos	864.1899	887.1792	C_45_H_36_O_18_	289.0714; 425.0902; 577.1345; 713.1506	Pavetannin B2 (Trimers of complex Proanthocyanidin A and B) [23]
		neg	864.1899	863.1867		125.0244; 289.0717; 423.0712; 575.1186; 711.1357	
	4	pos	866.2053	889.1945	C_45_H_38_O_18_	289.0706; 601.1310; 717.1510	Litchitannin B1 (Trimers of Proanthocyanidin B)
		neg	-	-		-	
P4	5	pos	-	-	C_30_H_24_O_12_	-	2 × PA(A) (Dimers of Proanthocyanidin A) [24]
		neg	576.1257	575.1184		-	
	6	pos	866.2056	867.2129	C_45_H_38_O_18_	247.0601; 289.0705; 427.1025; 579.1492; 715.1647	Litchitannin B2 (Trimers of Proanthocyanidin B)
		neg	866.2053	865.1981		125.0243; 287.0556; 407.0755; 575.1187; 713.1531	
	7	pos	864.1902	865.1974	C_45_H_36_O_18_	287.0550; 411.1073; 533.1076; 575.1179; 713.1497	3 × PA(A&B) (Trimers of complex Proanthocyanidin A and B) [23]
		neg	864.1899	863.1826		125.0247; 289.0716; 411.0720; 573.1025; 711.1346	
	8	pos	848.1951	849.2023	C_45_H_36_O_17_	287.0557; 517.1125; 713.1504	Trimeric PA (with an afzelechin or epiafzelechin) [23]
		neg	848.1948	847.1875		289.0710; 411.0723; 557.1095	
	9	pos	864.1905	865.1979	C_45_H_36_O_18_	287.0551; 425.0869; 577.1336; 713.1497	3 × PA (A&B) [23]
		neg	864.1899	863.1827		125.0243; 285.0404; 575.1201; 711.1362	
	10	pos	576.127	577.1342	C_30_H_24_O_12_	123.0440; 287.0549; 425.0864	Procyanidin A2 [25]

* The molecular formula and the compounds were deduced based on searching the *m/z* and MS/MS spectra in the Meltin database and compound information in the references. - For some compounds, whose negative or positive modes were not achived successfully.

**Table 2:** Primers for real-time PCR analysis.

Genes	GenBank ID	Forward Primer (5′ to 3′)	Reverse Primer (5′ to 3′)
*LcCHI*	HQ402910	CGGAGTTTACTTGGAGGATGT	CAGTGACCTTCTCAGAGTATTG
*LcANS*	HQ402913	AGGAAGTTGGTGGTCTGGAAG	CCGTTGCTGAGGATTTCAATGGTG
*LcUFGT*	HQ402914	GCCACCAGCGGTTCCTAATA	ATGCCTCTGCTACTGCTACAATCT
*LcLAR*	Litchi_GLEAN_10054331	AGATTGGACGAACTCTACC	TTGAAACAATCATCCAACG
*LcANR*	Litchi_GLEAN_10049142	ATACATTTGTGCTGCTTTC	TCATAGGTTTCTTCAAGCC
*LcActin*	HQ615689	ACCGTATGAGCAAGGAAATCACTG	TCGTCGTACTCACCCTTTGAAATC

## Supplementary Materials

Supplementary materials can be found at www.mdpi.com/1422-0067/19/1/301/s1.

## Abbreviations

GAE	gallic acid equivalents
RE	rutin equivalents
CCK-8	cell counting Kit-8
EC	(−)-epicatechin
C	(+)-catechin
EGCG:	(−)-epigallocatechin gallate;
ECG	(−)-epicatechin-3-gallate
CG	(−)-catechin gallate
FRAP	Ferric reducing antioxidant power
DAA	day after anthesis
CHI	chalcone isomerase
ANS	anthocyanin synthase
UFGT	UDP-glucose: flavonoid 3-*O*-glucosyltransferase
ANR	anthocyanidin reductase
LAR	leuacoanthocyanidin reductase
SEM	standard error of mean
BHT	butylated hydroxytoluene

## References

[1] Bhoopat L., Srichairatanakool S., Kanjanapothi D., Taesotikul T., Thananchai H., Bhoopat T. (2011). Hepatoprotective effects of lychee (*Litchi chinensis* sonn.): A combination of antioxidant and anti-apoptotic activities. J. Ethnopharmacol..

[2] Brat P., Georgé S., Bellamy A., Chaffaut L.D. (2006). Daily polyphenol intake in France from fruit and vegetables1. J. Nutr..

[3] Ibrahim S.R.M., Mohamed G.A. (2015). *Litchi chinensis*: Medicinal uses, phytochemistry, and pharmacology. J. Ethnopharmacol..

[4] Xu X., Xie H., Hao J., Jiang Y., Wei X. (2011). Flavonoid glycosides from the seeds of litchi chinensis. J. Agric. Food Chem..

[5] Chen Y.B., Wu K.S., Gu Y., Chen J.Z. (2007). Research progress in the chemical constituents and pharmacological effects of lychee seeds. J. Infect. Tradit. Chin. Med..

[6] Chen H.B., Wang H.C., Zhuang L.J. The litchi research and development in China in 2010. Proceedings of the 2011 China National Workship on Litchi and Longan.

[7] Sarni-Manchado P., Le Roux E., Le Guernevé C., Lozano Y., Cheynier V. (2000). Phenolic composition of litchi fruit pericarp. J. Agric. Food Chem..

[8] Duan X., Jiang Y., Su X., Zhang Z., Shi J. (2007). Antioxidant properties of anthocyanins extracted from litchi (*Litchi chinenesis* sonn.) fruit pericarp tissues in relation to their role in the pericarp browning. Food Chem..

[9] Zhao M., Yang B., Wang J., Liu Y., Yu L., Jiang Y. (2007). Immunomodulatory and anticancer activities of flavonoids extracted from litchi (*Litchi chinensis* sonn.) pericarp. Int. Immunopharmacol..

[10] Ogasawara J., Kitadate K., Nishioka H., Fujii H., Sakurai T., Kizaki T., Izawa T., Ishida H., Ohno H. (2009). Oligonol, a new lychee fruit-derived low-molecular form of polyphenol, enhances lipolysis in primary rat adipocytes through activation of the ERK1/2 pathway. Phytother. Res..

[11] Park C.H., Noh J.S., Fujii H., Roh S.-S., Song Y.-O., Choi J.S., Chung H.Y., Yokozawa T. (2015). Oligonol, a low-molecular-weight polyphenol derived from lychee fruit, attenuates gluco-lipotoxicity-mediated renal disorder in type 2 diabetic db/db mice. Drug Discov. Ther..

[12] Kessy H., Hu Z., Zhao L., Zhou M. (2016). Effect of steam blanching and drying on phenolic compounds of *Litchi pericarp*. Molecules.

[13] Singh S., Sharma B., Kanwar S.S., Kumar A. (2016). Lead phytochemicals for anticancer drug development. Front. Plant Sci..

[14] Zhang Z., Pang X., Yang C., Ji Z., Jiang Y. (2004). Purification and structural analysis of anthocyanins from *Litchi pericarp*. Food Chem..

[15] Hu Y., Deng L., Chen J., Zhou S., Liu S., Fu Y., Yang C., Liao Z., Chen M. (2016). An analytical pipeline to compare and characterise the anthocyanin antioxidant activities of purple sweet potato cultivars. Food Chem..

[16] Sommella E., Pepe G., Pagano F., Conte G., Carimi F., Tenore G.C., Novellino E., Manfra M., Russo M., Campiglia P. (2016). Rapid screening of antioxidant anthocyanins in autochthonous nero d’avola grape clones by pre-column DPPH reaction coupled to UHPLC-UV/Vis-IT-Tof: A strategy to combine chemical data and genetic diversity. Food Anal. Methods.

[17] Faramarzi S., Pacifico S., Yadollahi A., Lettieri A., Nocera P., Piccolella S. (2015). Red-fleshed apples: Old autochthonous fruits as a novel source of anthocyanin antioxidants. Plant Food Hum. Nutr..

[18] Faria A., Pestana D., Teixeira D., de Freitas V., Mateus N., Calhau C. (2010). Blueberry anthocyanins and pyruvic acid adducts: Anticancer properties in breast cancer cell lines. Phytother. Res..

[19] Wei Y.Z., Hu F.C., Hu G.B., Li X.J., Huang X.M., Wang H.C. (2011). Differential expression of anthocyanin biosynthetic genes in relation to anthocyanin accumulation in the pericarp of litchi chinensis sonn. PLoS ONE.

[20] Zhang D., Quantick P.C., Grigor J.M. (2000). Changes in phenolic compounds in litchi (*Litchi chinensis* sonn.) fruit during postharvest storage. Postharvest Biol. Technol..

[21] Jing G., Huang H., Yang B., Li J., Zheng X., Jiang Y. (2013). Effect of pyrogallol on the physiology and biochemistry of litchi fruit during storage. Chem. Cent. J..

[22] Zhao M., Yang B., Wang J., Li B., Jiang Y. (2006). Identification of the major flavonoids from pericarp tissues of lychee fruit in relation to their antioxidant activities. Food Chem..

[23] Xu X., Xie H., Wang Y., Wei X. (2010). A-type proanthocyanidins from lychee seeds and their antioxidant and antiviral activities. J. Agric. Food Chem..

[24] Wen L., Wu D., Jiang Y., Prasad K.N., Lin S., Jiang G., He J., Zhao M., Luo W., Yang B. (2014). Identification of flavonoids in litchi (*Litchi chinensis* sonn.) leaf and evaluation of anticancer activities. J. Funct. Foods.

[25] Liu L., Xie B., Cao S., Yang E., Xu X., Guo S. (2007). A-type procyanidins from litchi chinensis pericarp with antioxidant activity. Food Chem..

[26] Takanashi K., Suda M., Matsumoto K., Ishihara C., Toda K., Kawaguchi K., Senga S., Kobayashi N., Ichikawa M., Katoh M. (2017). Epicatechin oligomers longer than trimers have anti-cancer activities, but not the catechin counterparts. Sci. Rep..

[27] Chen Q., Zhang X.N., Yu H.W., Yan W., Tang H.R. (2012). Changes of total anthocyanins and proanthocyanidins in the developing blackberry fruits. J. Int. ChemTech Res..

[28] Carbone F., Preuss A., DeVos R.C.H., D’Amico E., Perrotta G., Bovy A.G., Martens S., Rosati C. (2009). Developmental, genetic and environmental factors affect the expression of flavonoid genes, enzymes and metabolites in strawberry fruits *. Plant Cell Environ..

[29] Bogs J., Jaffé F.W., Takos A.M., Walker A.R., Robinson S.P. (2007). The grapevine transcription factor vvmybpa1 regulates proanthocyanidin synthesis during fruit development. Plant Physiol..

[30] Wang Y., Li J., Xia R. (2010). Expression of chalcone synthase and chalcone isomerase genes and accumulation of corresponding flavonoids during fruit maturation of guoqing no. 4 satsuma mandarin (*Citrus unshiu* Marcow). Sci. Horticult..

[31] Guan C., Song X., Ji J., Li X., Jin C., Guan W., Li J., Wang G. (2014). Salicylic acid treatment enhances expression of chalcone isomerase gene and accumulation of corresponding flavonoids during fruit maturation of lycium chinense. Eur. Food Res. Technol..

[32] Wang H., Wang W., Li H., Zhang P., Zhan J., Huang W. (2011). Expression and tissue and subcellular localization of anthocyanidin synthase (ANS) in grapevine. Protoplasma.

[33] Szankowski I., Flachowsky H., Li H., Halbwirth H., Treutter D., Regos I., Hanke M.-V., Stich K., Fischer T.C. (2009). Shift in polyphenol profile and sublethal phenotype caused by silencing of anthocyanidin synthase in apple (*Malus* sp.). Planta.

[34] Zhang J., Han Z.Y., Tian J., Zhang X., Song T.T., Yao Y.C. (2015). The expression level of anthocyanidin synthase determines the anthocyanin content of crabapple (*Malus* sp.) petals. Acta Physiol. Plant..

[35] Yoshihara N., Imayama T., Fukuchi-Mizutani M., Okuhara H., Tanaka Y., Ino I., Yabuya T. (2005). cDNA cloning and characterization of UDP-glucose: Anthocyanidin 3-*O*-glucosyltransferase in iris hollandica. Plant Sci..

[36] Lai B., Li X.J., Hu B., Qin Y.H., Huang X.M., Wang H.C., Hu G.B. (2014). LcMYB1 is a key determinant of differential anthocyanin accumulation among genotypes, tissues, developmental phases and aba and light stimuli in litchi chinensis. PLoS ONE.

[37] Tanner G.J., Francki K.T., Abrahams S., Watson J.M., Philip J., Larkin P.J., Ashton A.R. (2003). Proanthocyanidin biosynthesis in plants. Purification of legume leucoanthocyanidin reductase and molecular cloning of its cDNA. J. Biol. Chem..

[38] Xie D.Y., Sharma S.B., Paiva N.L., Ferreira D., Dixon R.A. (2003). Role of anthocyanidin reductase, encoded by BANYULS in plant flavonoid biosynthesis. Science.

[39] Pfeiffer J., Kühnel C., Brandt J., Duy D., Punyasiri P.A.N., Forkmann G., Fischer T.C. (2006). Biosynthesis of flavan 3-ols by leucoanthocyanidin 4-reductases and anthocyanidin reductases in leaves of grape (*Vitis vinifera* L.), apple (*Malus x domestica* Borkh.) and other crops. Plant Physiol. Biochem..

[40] Zhang X.N., Chen Q., Yu D.Q., Zhou S.L., Tang H.R. (2013). Expression analysis of the ANR and LAR gene in *Fragaria* × *ananassa* cv. Toyonaka. J. Agric. Sci..

[41] Gagné S., Lacampagne S., Claisse O., Gény L. (2009). Leucoanthocyanidin reductase and anthocyanidin reductase gene expression and activity in flowers, young berries and skins of *Vitis vinifera* L. Cv. Cabernet-sauvignon during development. Plant Physiol. Biochem..

[42] Dewanto V., Wu X., Adom K.K., Liu R.H. (2002). Thermal processing enhances the nutritional value of tomatoes by increasing total antioxidant activity. J. Agric. Food Chem..

[43] Zhishen J., Mengcheng T., Jianming W. (1999). The determination of flavonoid contents in mulberry and their scavenging effects on superoxide radicals. Food Chem..

[44] Benzie I.F.F., Strain J.J. (1996). The ferric reducing ability of plasma (FRAP) as a measure of “antioxidant power”: The frap assay. Anal. Biochem..

[45] Brand-Williams W., Cuvelier M.E., Berset C. (1995). Use of a free radical method to evaluate antioxidant activity. LWT Food Sci. Technol..

[46] Zheng W.J., Feng Q., Liu J., Guo Y.K., Gao L.F., Li R.M., Xu M., Yan G.Z., Yin Z.N., Zhang S. (2017). Inhibition of 6-phosphogluconate dehydrogenase reverses cisplatin resistance in ovarian and lung cancer. Front. Pharmacol..

[47] Rzeppa S., Von Bargen C., Bittner K., Humpf H.U. (2011). Analysis of flavan-3-ols and procyanidins in food samples by reversed phase high-performance liquid chromatography coupled to electrospray ionization tandem mass spectrometry (RP-HPLC-ESI-MS/MS). J. Agric. Food Chem..

[48] Ponchel F., Toomes C., Bransfield K., Leong F.T., Douglas S.H., Field S.L., Bell S.M., Combaret V., Puisieux A., Mighell A.J. (2003). Real-time PCR based on SYBR-green I fluorescence: An alternative to the taqman assay for a relative quantification of gene rearrangements, gene amplifications and micro gene deletions. BMC Biotechnol..

