# Supplementary material for: B Type and Complex A/B Type Epicatechin Trimers Isolated from Litchi pericarp Aqueous Extract Show High Antioxidant and Anticancer Activity
Source: Int J Mol Sci. 2018 Jan 19;19(1):301. doi: 10.3390/ijms19010301 (PMC5796246; doi:10.3390/ijms19010301)
Supplement: Supplementary file 1 [file ijms-19-00301-s001.pdf]

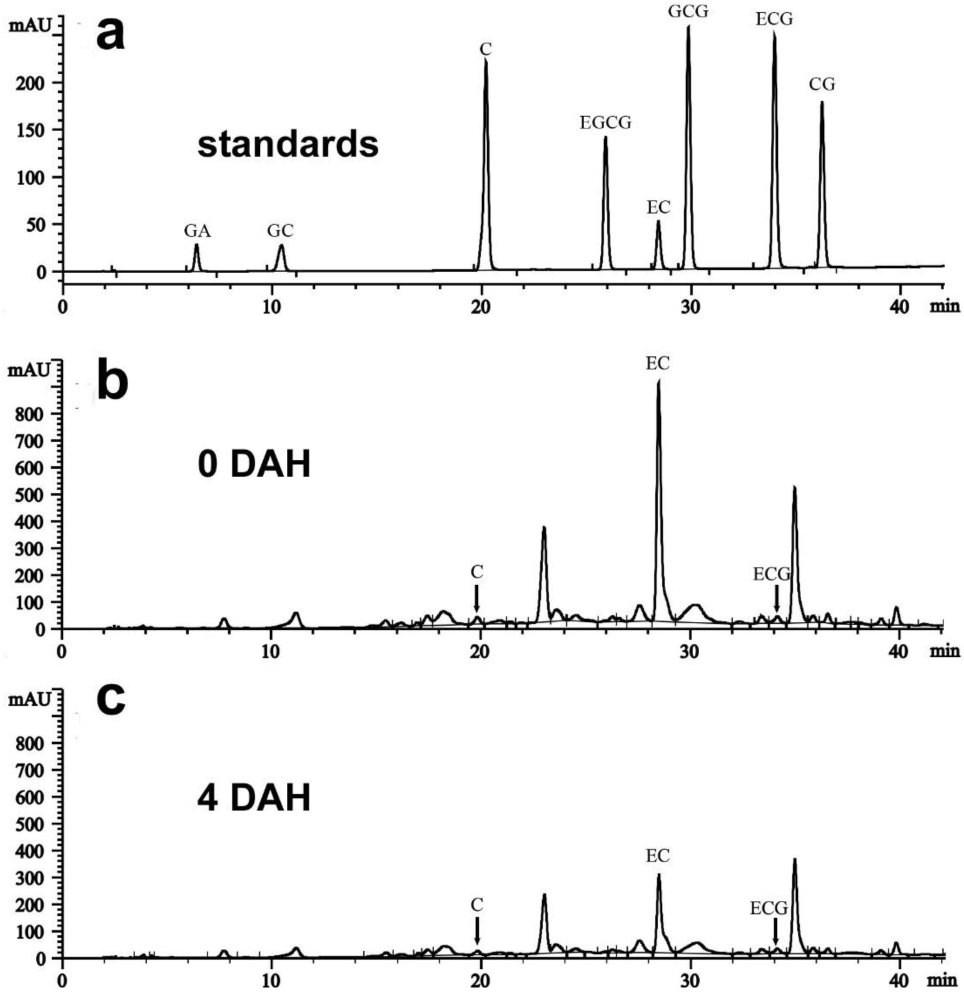

Figure S1. Analysis of catechin-type compounds in the Litchi pericarp by HPLC during fruit storage after harvest. (a) HPLC separation of the standards of catechin-type compounds. HPLC profiles of the catechin-type compounds in the Litchi pericarp of the mature fruit at 0 day after harvest (DAH) and the fruit stored 4 DAH at 20°C were shown in (b) and (c). EC and C were identified in the samples as described in Figure S1.
